# Lanosterol Synthase Pathway Alleviates Lens Opacity in Age-Related Cortical Cataract

**DOI:** 10.1155/2018/4125893

**Published:** 2018-07-11

**Authors:** Xinyue Shen, Manhui Zhu, Lihua Kang, Yuanyuan Tu, Lele Li, Rutan Zhang, Bai Qin, Mei Yang, Huaijin Guan

**Affiliations:** ^1^Department of Ophthalmology, Affiliated Hospital of Nantong University, Nantong, Jiangsu 226001, China; ^2^Department of Ophthalmology, Wuxi No. 3 People's Hospital, Wuxi, Jiangsu 214041, China; ^3^Department of Ophthalmology, Lixiang Eye Hospital of Soochow University, Suzhou 215021, China; ^4^Department of Chemistry, Fudan University, Shanghai 200433, China

## Abstract

**Purpose:**

Lanosterol synthase (LSS) abnormity contributes to lens opacity in rats, mice, dogs, and human congenital cataract development. This study examined whether LSS pathway has a role in different subtypes of age-related cataract (ARC).

**Methods:**

A total of 390 patients with ARC and 88 age-matched non-ARC patients were enrolled in this study. LSS expression was analyzed by western blot and enzyme-linked immunosorbent assay (ELISA). To further examine the function of LSS, we used U18666A, an LSS inhibitor in rat lens culture system.

**Results:**

In lens epithelial cells (LECs), LSS expression in LECs increased with opaque degree C II, while it decreased with opaque degree C IV and C V. While in the cortex of age-related cortical cataract (ARCC), LSS expression was negatively related to opaque degree, while lanosterol level was positively correlated to opaque degree. No obvious change in both LSS and lanosterol level was found in either LECs or the cortex of age-related nuclear cataract (ARNC) and age-related posterior subcapsular cataract (ARPSC). In vitro, inhibiting LSS activity induced rat lens opacity and lanosterol effectively delayed the occurrence of lens opacity.

**Conclusions:**

This study indicated that LSS and lanosterol were localized in the lens of human ARC, including ARCC, ARNC, and ARPSC. LSS and lanosterol level are only correlated with opaque degree of ARCC. Furthermore, activated LSS pathway in lens is protective for lens transparency in cortical cataract.

## 1. Introduction

Cataract is one of the leading causes of blindness worldwide and cataract is the major cause of blindness in developing countries, including China [1, 2]. The clinical characteristic of cataract is lenticular opacity and the final visual impairment. Age-related cataract (ARC) is the most common type [3, 4]. According to different region of opacity, ARC is classified into three subtypes: age-related cortical cataract (ARCC), age-related nuclear cataract (ARNC), and age-related posterior subcapsular cataract (ARPSC) [5]. Currently, the only cure for cataract is surgical removal of the opaque lenses and substitution with transparent artificial intraocular lenses [6]. Preventing or delaying the onset of cataracts by pharmacological interventions has been an attractive field of research in ophthalmology [7, 8].

The reason for the relative absence of cataract drugs is that the etiology for cataract remains unclear. Studies have identified multiple risk factors related to cataract formation, including genetic predisposition, aging, oxidative stress, ultraviolet (UV) light, systematic diseases, and toxic agents [9, 10]. Lipid metabolic disorders, especially cholesterol metabolic disturbance, have been proposed to be a potential factor for cataract. However, whether cholesterol is a risk or protective factor in cataract remains in debate [11–13]. Recently, researchers demonstrate that lanosterol, not cholesterol, plays a preventive role in cataract formation, inhibiting lens opacity, and reversing crystalline aggregation [14]. However, the relationship between lanosterol and the subtypes of ARC is unknown.

Lanosterol is the first sterol in lipid biosynthetic pathway, which is initially converted by acetyl-CoA. The complex process of lanosterol synthesis involves several enzymes, including 3-hydroxy-3-methylglutaryl-coenzyme A (HMG-CoA) reductase, squalene epoxidase, and lanosterol synthase (LSS). LSS is a microsomal enzyme that functions as a downstream element in the lanosterol biosynthetic pathway [15], catalyzing the cyclization of the linear 2,3-monoepoxysqualene to lanosterol [15]. Inhibiting LSS activity can induce cataract in rats, dogs, and mice both in vitro and in vivo [16, 17]. Shumiya cataract rat (SCR) is a hereditary cataractous rat strain that develops cataract at approximate 11 weeks of age [18]. *Lss* gene is mutated in SCR and is the major gene for cataract onset [19]. In humans, two distinct homozygous LSS missense mutations (W581R and G588S) happen in two families with extensive congenital cataracts, which impairs key catalizing functions of LSS. [14]. However, whether LSS pathway plays a role in ARC remains obscure.

In the present study, the expression of LSS, the key enzyme in the synthesis of lanosterol, in the human lens tissue was measured and the relationship between lanosterol, LSS level, and opaque degree was analyzed. Additionally, in an in vitro whole rat lens culture model, U18666A (an LSS inhibitor) was used to determine the causative effect of LSS activity on lens opacity, which may produce a novel potential strategy for the therapy of cataract.

## 2. Materials and methods

### 2.1. Human Lens Extracts

Human lens samples including lens capsules (mainly composed of LECs) and the cortex were obtained from Department of Ophthalmology, Affiliated Hospital of Nantong University. The lens tissues from 390 patients with different subtypes of ARC during phacoemulsification were collected. The clinical diagnosis of ARC is based on criteria of the Lens Opacities Classification System II (LOCS II) [20]. We also included 88 age-matched individuals without cataract but had their lens extracted because of vitreoretinal diseases as control during extracapsular cataract extraction. Patients with the following conditions were excluded: other eye diseases such as high myopia, dislocated lens, glaucoma, uveitis, and diabetic retinopathy and systemic diseases such as hepatic dysfunction, diabetes, cancer, uncontrolled hypertension, hyperlipemia, obstructive sleep apnea, and nervous system diseases such as Alzheimer's disease. All of the extracts were preserved in −80°C until further analysis. Ethical permission was obtained from the Medical Ethics Committee of Affiliated Hospital of Nantong University (No. 2012(021)), and the informed consent was received from all patients according to the tenets of the Declaration of Helsinki.

### 2.2. Rat Lens Extracts

96 male Sprague Dawley rats (SD rats) at approximately 4 weeks of age, weighing 150–180 g with normal lens were provided from the Laboratory Animal Center of Nantong University. Rats were housed in solid bottom cages in an environmentally controlled room (20 ± 2°C, 50 ± 15% humidity) on a 12 h light/dark cycle and freely accessed standard laboratory chow and water. Before extracting lens, the rats were anesthetized by intraperitoneal injection of 80 mg/kg pentobarbital sodium. The eye globes were collected immediately after sacrifice. All experimental procedures were performed in accordance with the requirements of Animal Care and Use Committee of Nantong University.

### 2.3. Western Blot

The protein of human LECs, cortex, and rat lens in each group were extracted in RIPA lysis buffer III (Sangon Biotech, China) as per the instructions. After ultrasonication, the specimens were centrifuged at 12000 rpm (15 min, 4°C), and the supernatant was collected. Equal amounts of lysates (100 *μ*g/lane) were separated on 10% SDS-PAGE and transferred to PVDF membranes. After blocking in 5% skimmed milk (TBST as vehicle) for 2 h, the membranes were incubated with rabbit anti-LSS antibody (1 : 500, Abcam, UK) and mouse anti-GAPDH antibody (1 : 5000, ABclonal, France) overnight at 4°C. After washing, the membranes were incubated with goat HRP-conjugated secondary antibodies (Jackson ImmunoResearch, USA). Proteins were visualized using an ECL system (Cell Signaling, USA). Relative level of proteins was quantified with ImageJ software (National Institutes of Health, USA).

### 2.4. Enzyme-Linked Immunosorbent Assay (ELISA)

The LSS level of human LECs and the cortex of cultured lens were quantified by ELISA using human LSS ELISA kit (QIYBO, China) according to the manufacturer's instructions. The optical density was read at 450 nm wavelength using a microplate reader (ELx800, BioTek, USA).

### 2.5. Lens Explant Culture and Reagent

The lenses extracted from the globes were cultured in M199 medium (Gibco, Rockville, MD) supplemented with 100 U/ml penicillin and 0.1 mg/ml streptomycin in a 75% humidified 37°C incubator with 21% O_2_ and 5% CO_2_ for 1 day in 24 well cell culture clusters. Lenses without opacification were then randomized into five groups with 3 lenses or more per treatment: untreated control, vehicle (DMSO) control, U18666A treatment, lanosterol treatment, and U18666A and lanosterol treatment. U18666A solution was prepared using DMSO as the solvent with the final concentration of 200 nM in culture media. Lanosterol solution was prepared using M199 medium with the final concentration of 40 *μ*M. Final medium osmolality of each group was maintained from 295 to 310 mOsM/kg. The medium was replaced every 24 h. The opacity of lens was image analyzed at 1, 3, and 5 days. At the end of culture, the lenses were weighed and preserved in −80°C for further experiments.

### 2.6. Lens Transparency Assessment

For imaging, an antibody incubation box which had been high-pressure steam disinfected was placed in vertical clean bench. To observe the transparency of lens, we sprayed 75% alcohol on vertical clean bench as much as possible. 24 well cell culture clusters were taken from incubator and placed on the antibody incubation box carefully, making sure there were no air bubbles but only 75% alcohol between the clusters and antibody incubation box. The image was captured with the Nikon D90 camera under the largest magnification. In this way, the transparency of total lens area was observed, with the black image representing transparent lens, gray image representing opaque lens.

### 2.7. Immunofluorescence Assay

The cultured rat lens was fixed with chloroform fixative overnight and sugar dehydrated followed by frozen section. After permeabilization and blocking in 3% fetal bovine serum with 0.3% TritonX-100 for 30 min, the sections were incubated overnight at 4°C with IgG (negative control), anti-LSS antibody (1 : 500, Abcam, UK), and anti-KDEL antibody (1 : 250, Abcam, UK). After washing, tissue sections were incubated with FITC-conjugated (1 : 250) or Cy3-conjugated secondary antibody (1:1000) (Jackson ImmunoResearch) for 2 h at 37°C.

### 2.8. Statistical Analysis

All data were presented as means ±SD. SPSS software (SPSS 17.0; SPSS Inc., USA) was used for statistical analysis. One-way ANOVA was used for statistical comparisons of multiple groups. The difference between two groups was determined by Student's *t*-test. Statistical significance was based on a *P* value < 0.05. Each experiment consisted of at least three replicates.

## 3. Results

### 3.1. LSS and Lanosterol Level Changes in LECs and Cortex of Human ARCC

**Figure 1:**
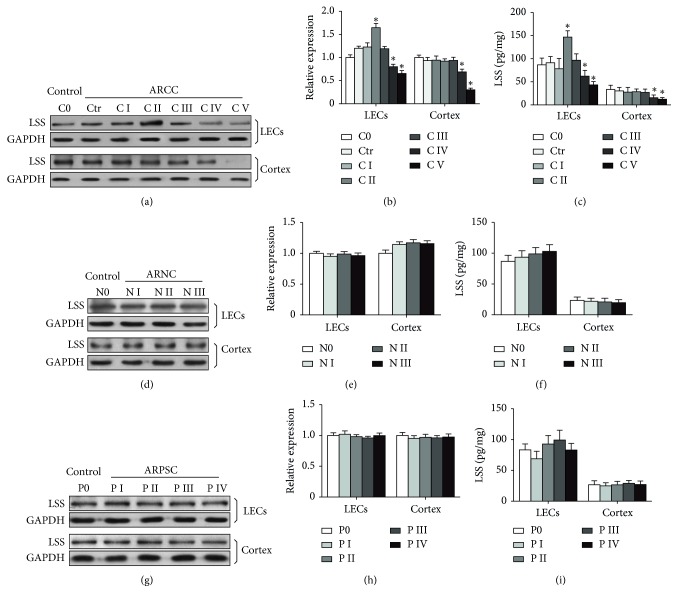
Protein levels of LSS in the human LECs and the cortex with different opaque degree from age-related cortical cataract (ARCC), age-related nuclear cataract (ARNC), age-related posterior subcapsular cataract (ARPSC). (a, d, g) LECs of control and ARCC, ARNC, ARPSC patients were processed for western blot with antibody against LSS and GAPDH (*n*=15 for each subtype), respectively. (b, e, h) Quantification of LSS protein levels normalized to GAPDH from western blot in (a), (d), and (g), respectively. (c, f, i) LSS protein levels (pg/mg protein) in ARCC, ARNC, ARPSC patients measured by ELISA (*n*=15 for each subtype). ^*∗*^*P* < 0.05.

To detect LSS level in LECs and the cortex in human ARC tissues, western blot was performed, showing that LSS changed along with the cataract levels in LECs of human ARCC, increasing at C II and decreasing at C IV and C V. While in the cortex of human ARCC, LSS decreased with the aggravation of opaque degree (Figures 1 and 1). Furthermore, ELISA showed that LSS had the similar change trends in different types of human ARC tissues (Figure 1). However, there were no significant changes of LSS level in either LECs or the cortex of human ARNC and ARPSC (Figures 1–1).

**Figure 2:**
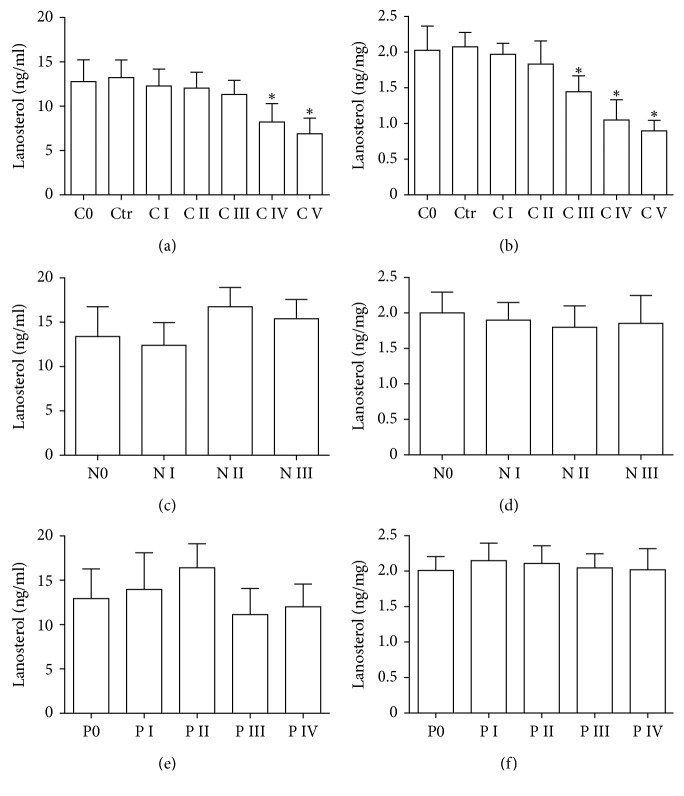
Lanosterol levels in the human LECs and cortex of different subtypes of ARC. (a, b, e). Lanosterol levels in LECs of with different opaque degree from ARCC, ARNC, and ARPSC (*n* = 15 for each subtype), respectively. (b, d, f). Lanosterol levels in cortex of with different opaque degree from ARCC, ARNC, and ARPSC (*n*= 15 for each subtype). ^*∗*^*P* < 0.05.

Lanosterol, the catalyst of LSS, was down-regulated in LECs and the cortex of human ARCC, especially in C IV and C V groups, decreasing in LECs from C IV to C V and in the cortex from C III till C V (Figures 2 and 2). However, lanosterol changed slightly in LECs and the cortex of human ARNC and ARPSC (Figures 2(c)–2(f)). The data were consistent with the results of LSS level, thus ARCC was chosen for subsequent experiments.

### 3.2. LSS Pathway Delays Lens Opacity

**Figure 3:**
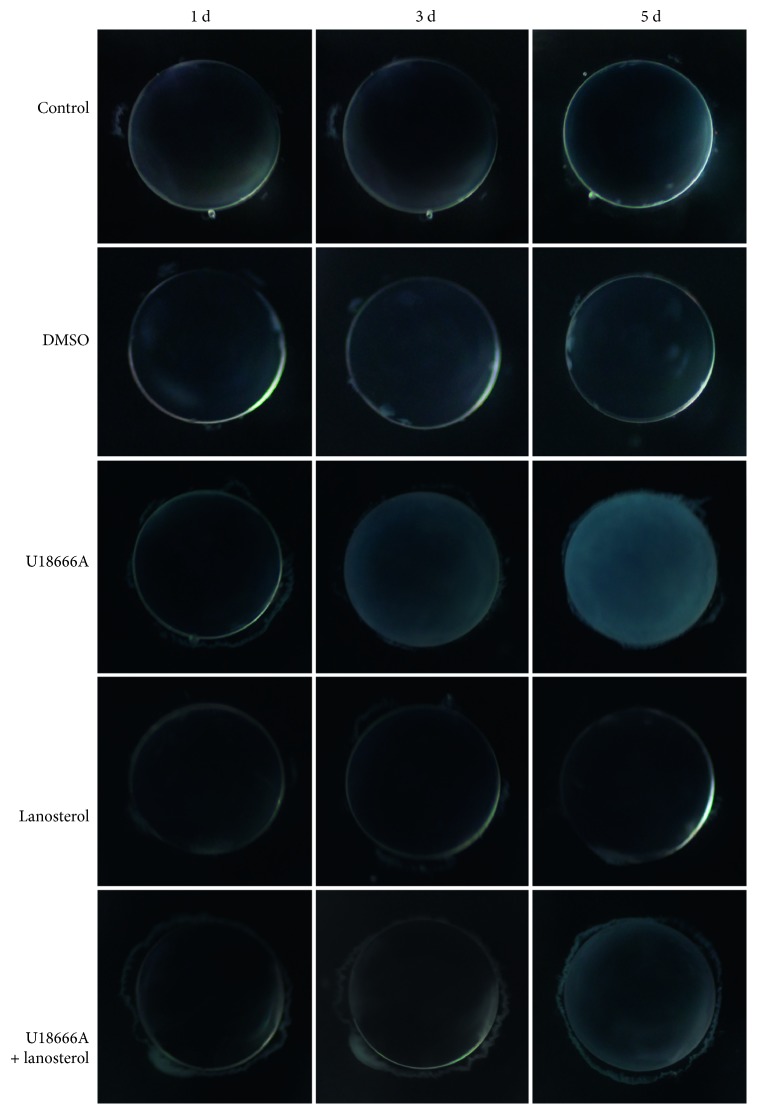
Images of the representative rat lens in each group taken in 5 days. Lens were cultured with DMSO (vehicle), 200 nM U18666A, 40 *μ*M lanosterol, or both U18666A and lanosterol for 5 days.

**Figure 4:**
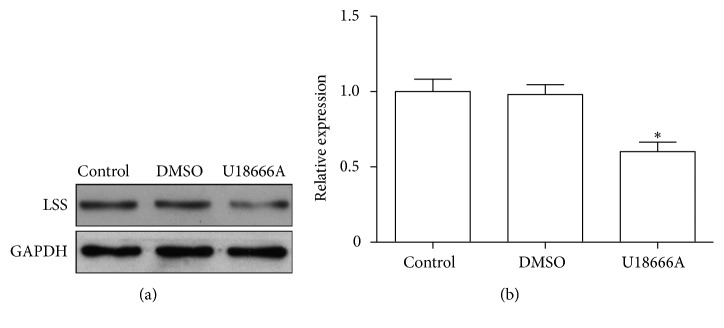
Protein levels of the LSS in rat lens in each group. (a) Rat lens were processed for western blot with antibody against LSS and GAPDH (*n*=5). (b) Quantification of LSS protein levels normalized to GAPDH from western blot in (a). ^*∗*^*P* < 0.05.

**Figure 5:**
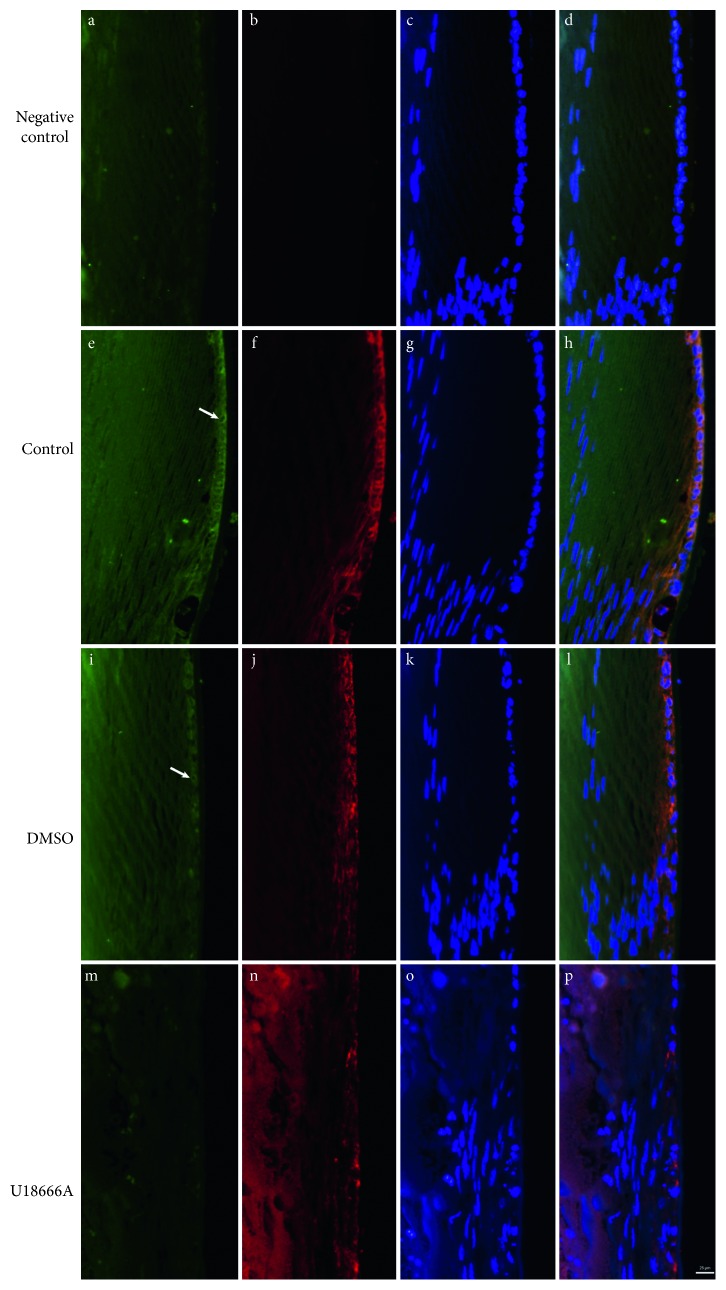
LSS localization in cultured rat lens in each group (*n*=3). The image is shown at ×20 magnification. Black: IgG (a, b); green: LSS (e, i, m); red: KDEL (f, j, n); blue: Hoechst (c, g, k, o); black: merge (d, h, l, p). The hollow arrow shows the LSS location.

To further identify the function of LSS pathway in the formation of ARCC, LSS inhibitor U18666A was used in lens culture system in vitro. U18666A treatment adversely affected the transparency of explanted rat lens within 3 d of exposure, indicated by blurring in the cortical area, while lanosterol effectively delayed the occurrence of lens opacity, but not to prevent opacity (Figure 3). After U18666A treatment, LSS expression decreased in opaque rat lens in comparison to transparent ones (Figures 4(a) and 4(b)). LSS is a membrane-associated enzyme, which is targeted to the cytoplasmic leaflet of endoplasmic reticulum (ER) [21]. Immunostaining showed colocalization of LSS with Lys-Asp-Glu-Leu (KDEL; an ER marker, ER retention sequence) [22] and the level of LSS markedly decreased after U18666A treatment (Figure 5).

**Figure 6:**
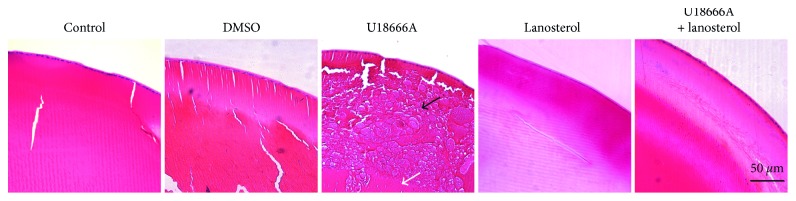
Microscopy of rat lenses treated with U18666A (*n*=3). The image is shown at ×20 magnification. The hollow arrow shows regularly arranged fibers in nuclei. The black arrow shows fiber degeneration in the cortex.

Microscopy showed that there was a breakdown of structural integrity with extensive disruption in the cortical fiber cells of the opaque lens following U18666A treatment. This disrupted cortical region was completely different from nuclear region, where the fiber cells remained in order. Lens treated with lanosterol suggested slight fracture in the cortex (Figure 6).

## 4. Discussion

No medical treatment has any effect on cataract for its unknown etiology. Lipid metabolic disorder in cataract has recently emerged as an important field of investigation. Decreased LSS level has been observed in both SCR and human congenital cataract [14, 19], yet LSS in human ARC has not been studied. In our study, LSS expression increased when the lens was slightly opaque and then decreased when the lens were seriously opaque in LECs. In addition, LSS expression decreased in the cortex of human ARCC with the increase of damage level. In vitro, inhibiting LSS induced rat lens opacity in the cortex in 3 days. We further showed that restoring lanosterol level delayed the occurrence of opacity. These data suggest that LSS and lanosterol change with the increase of opacity of lens in ARCC, indicating that LSS pathway plays a protective role in cortical cataract.

In our study, we are unable to draw a conclusion that LSS pathway plays a protective role in human ARCC as we could not obtain the whole human lens by the process of phacoemulsification. Accordingly, we used whole rat lens to keep the complete lens structure. Recent research shows that lanosterol solution does not reverse opacification of human age-related cataractous nuclei, using human cataractous nuclei instead of whole human ARC lens [23]. Given the different structure between the cortex and nuclei, as well as lacking the intact structure, it cannot be concluded that lanosterol cannot reverse opacification of human ARC. Therefore, whether LSS pathway plays a protective role in human ARCC needs further investigation.

Intriguingly, the change of LSS is different in LECs and the cortex. As the relatively simple cellular structure compared with other parts of the eye, LECs are the fundamental protection responsible for normal occurrence and growth of transparent lens [24]. The lens epithelium is also the initiation site for many other types of cataract [25–28]. In our study, the level of LSS increased in LECs when the opaque degree was C II with no accumulation of lanosterol and no prevention of aggravation of opacity. It is therefore reasonable to assume that LECs can synthesize more LSS without more lanosterol just as a stress response. While the mature fiber cells lose their nuclei and other cytosolic organelles during differentiation and their ability to synthesize LSS, LSS in the cortex decreases when the lens becomes opaque. Therefore, we assume that the increased LSS in LECs might fill another function rather than just being an enzyme catalyzing lanosterol synthesis [15]. Actually, apart from catalyzing lanosterol synthesis, LSS also catalyzes 24(S), 25-epoxylanosterol synthesis from 2, 3: 22, 23-diepoxysqualene, which regulates the liver X receptor, whose relationship with cataract has not been well explored.

Although risk factors accounting for ARC are varied, oxidative stress is well acknowledged. In response to oxidative stress, lens proteins including enzyme and crystallins become modified, denatured, and aggregated, contributing to cataract formation [29]. Oxidative stress alters enzymatic activities and cell proliferation. Why LSS protects lens transparency is unclear, but its role in nervous system has been extensively studied. Previous study has implied that inhibition of LSS causes secondary apoptosis in neurons due to increase in reactive oxygen species (ROS) [30]. It also observed a decreased catalase (CAT) and superoxide dismutase (SOD) activity in astrocytes after inhibiting LSS activity, which causes the dysfunction of lipid metabolism in the cerebral cortex [31, 32]. As a result, we speculate that LSS resists oxidation and after inhibiting LSS activity, oxidative stress increases and leads to dysfunctional lipid metabolism. However, our data showed no association between LSS activity and ARNC or ARPSC, which might due to other molecular mechanisms causing lens opacity in these two subtypes.

## 5. Conclusion

LSS and lanosterol expression change in LECs and the cortex of human ARCC. In addition, a sustained LSS pathway delays the occurrence of rat lens cortical opacity in vitro, which may make sense to clinical pharmacotherapy of cataract in the future.

## Data Availability

The data used to support the findings of this study are available from the corresponding author upon request.

## Abbreviations

LSS: Lanosterol synthase
ARC: Age-related cataract
LC-MS: Liquid chromatograph-mass spectrometer
ARCC: Age-related cortical cataract
LECs: Lens epithelial cells
ARNC: Age-related nuclear cataract
ARPSC: Age-related posterior subcapsular cataract;
SCR: Shumiya cataract rat
LOCS II: Lens Opacities Classification System II
UPLC: Ultra performance liquid chromatograph
APCI: Atmospheric pressure chemical ionization source in position mode
MRM: Multiple reaction monitoring
ER: Endoplasmic reticulum.
